# Supplementary material for: A Global Screen for Assembly State Changes of the Mitotic Proteome by SEC-SWATH-MS
Source: Cell Syst. 2020 Feb 26;10(2):133–155.e6. doi: 10.1016/j.cels.2020.01.001 (PMC7042714; doi:10.1016/j.cels.2020.01.001)

O75815 | BCAR3_HUMAN | BCAR3 NSP2 SH2D3B UNQ271/PRO308
Monomer MW [kDa]: 92.566 Monomer expected elution fraction: 41

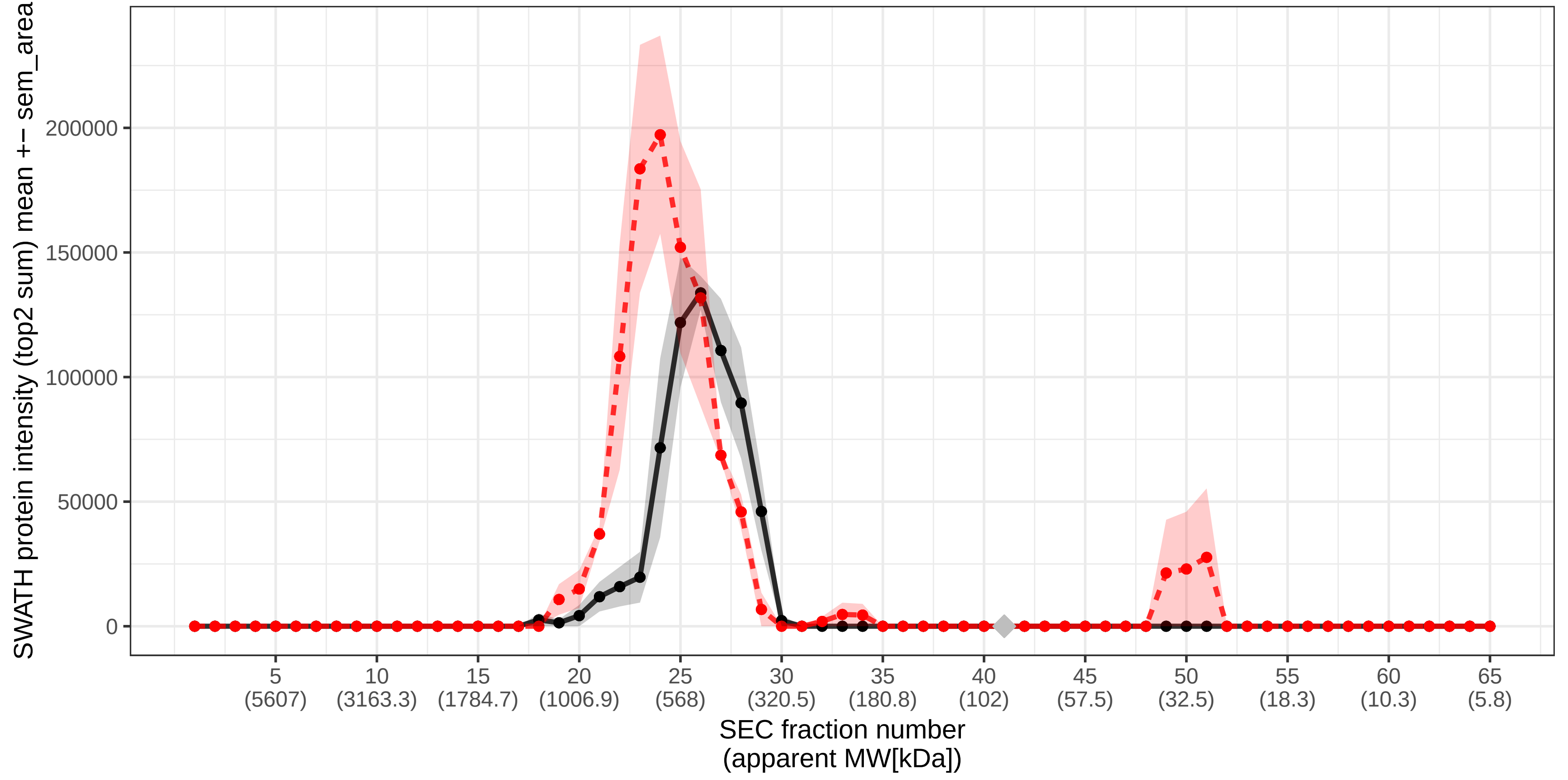

Supplement: Data S1. SEC-SWATH-MS Protein Chromatograms, Related to Figure 1 [file mmc6.zip › SECchrom_O75815_BCAR3_HUMAN_BCAR3_NSP2_SH2D3B_UNQ271_PRO308.pdf]
